# Emerging insights of tumor heterogeneity and drug resistance mechanisms in lung cancer targeted therapy

**DOI:** 10.1186/s13045-019-0818-2

**Published:** 2019-12-09

**Authors:** Zuan-Fu Lim, Patrick C. Ma

**Affiliations:** 1 WVU Cancer Institute, West Virginia University, Morgantown, WV 26506 USA; 2 Cancer Cell Biology Program, Robert C. Byrd Health Sciences Center, West Virginia University, Morgantown, WV 26506 USA; 3 Penn State Cancer Institute, Penn State Health Milton S. Hershey Medical Center, Penn State University, P.O. Box 850, Mail Code CH46, 500 University Drive, Hershey, PA 17033-0850 USA

**Keywords:** Tumor heterogeneity, Acquired drug resistance, Adaptive evolution, Minimal residual disease

## Abstract

The biggest hurdle to targeted cancer therapy is the inevitable emergence of drug resistance. Tumor cells employ different mechanisms to resist the targeting agent. Most commonly in *EGFR*-mutant non-small cell lung cancer, secondary resistance mutations on the target kinase domain emerge to diminish the binding affinity of first- and second-generation inhibitors. Other alternative resistance mechanisms include activating complementary bypass pathways and phenotypic transformation. Sequential monotherapies promise to temporarily address the problem of acquired drug resistance, but evidently are limited by the tumor cells’ ability to adapt and evolve new resistance mechanisms to persist in the drug environment. Recent studies have nominated a model of drug resistance and tumor progression under targeted therapy as a result of a small subpopulation of cells being able to endure the drug (minimal residual disease cells) and eventually develop further mutations that allow them to regrow and become the dominant population in the therapy-resistant tumor. This subpopulation of cells appears to have developed through a subclonal event, resulting in driver mutations different from the driver mutation that is tumor-initiating in the most common ancestor. As such, an understanding of intratumoral heterogeneity—the driving force behind minimal residual disease—is vital for the identification of resistance drivers that results from branching evolution. Currently available methods allow for a more comprehensive and holistic analysis of tumor heterogeneity in that issues associated with spatial and temporal heterogeneity can now be properly addressed. This review provides some background regarding intratumoral heterogeneity and how it leads to incomplete molecular response to targeted therapies, and proposes the use of single-cell methods, sequential liquid biopsy, and multiregion sequencing to discover the link between intratumoral heterogeneity and early adaptive drug resistance. In summary, minimal residual disease as a result of intratumoral heterogeneity is the earliest form of acquired drug resistance. Emerging technologies such as liquid biopsy and single-cell methods allow for studying targetable drivers of minimal residual disease and contribute to preemptive combinatorial targeting of both drivers of the tumor and its minimal residual disease cells.

## Introduction

New technologies and analyses in genomics have paved the way for a paradigm shift in the diagnostics, classification, and treatment of many cancer types [1–4] including lung cancer [5, 6]. The identification of actionable oncogenic mutations has greatly improved the treatment of various human cancers, as evident by the development and approved clinical use of many molecularly targeted therapeutics that can specifically target and inhibit driver mutations. In non-small cell lung cancers (NSCLCs), the discovery of activating mutations in the epidermal growth factor receptor (*EGFR*) gene such as the missense mutation L858R within exon 21 and short in-frame deletions within exon 19 have ushered in a new era of genomics-guided precision targeted therapy in lung cancer. These EGFR-targeted tyrosine kinase inhibitors (TKIs) such as erlotinib, gefitinib, and afatinib have shown improved tumor response and progression-free survival outcome in *EGFR*-mutated NSCLC compared with cytotoxic chemotherapies [7–9]. Other prominent examples of targeted therapies include kinase inhibitors of oncogenic receptor tyrosine kinases (RTKs) such as anaplastic lymphoma kinase (ALK), MET, ROS1, RET, and tropomyosin receptor kinase (TRK) as well as downstream target kinases such as BRAF. This phenomenon validated to a great extent the concept of “oncogene addiction” [10], in which tumors have grown to be dependent on the oncogenic activity of a single oncogene product to transform, proliferate, invade, and metastasize [11–14]. Even metastatic tumors that share similar characteristics with the primary tumor can respond remarkably to the same therapy [15, 16]. Harnessing the concept of oncogene addiction, genomics-guided targeted therapy has transformed the face of lung cancer treatment.

Despite great promises brought about by the new paradigm of cancer targeted therapy, various new challenges have proven paramount as well. The invariable emergence of acquired drug resistance not only limits the duration of tumor response but also represents the major obstacle for a more meaningful impact on long-term survival in genotype-matched precision medicine [17–19]. In both partial and complete responders, clinical drug resistance develops later in the course of therapy despite initial rapid and remarkable tumor regression [20], leading to therapeutic failure and ultimate patient demise [21–28]. Tumors can develop drug resistance during either the early phase or the late phase of drug treatment. Initial efforts in the studies of precision drug resistance focused on the two categories of therapeutic resistance: (1) intrinsic or primary resistance, and (2) acquired or secondary resistance. These are concepts that were essentially born from the fundamentals of clinical tumor response classification and assessment. Intrinsic drug resistance relates to a lack of initial tumor shrinkage upon precision therapy use. This phenomenon is thought to be mainly a result of tumor heterogeneity either within the tumor or among different tumor sites within a host. Concurrent non-target genomic aberrations may exist within driver-mutated or non-driver-mutated tumor cells that explain the lack of tumor response under the precision therapy targeting only one driver mutation. On the other hand, research in understanding the acquired drug resistance have largely focused on deciphering the molecular resistance mechanism in tumor tissues that have emerged as clinically progressing measurable disease. Typically, these studies emphasize on interrogating acquired drug resistance during the late phase of clinical treatment when the tumors progress as new metastatic lesions or as proliferation of previously responsive preexisting tumor lesions, and become clinically evident on imaging studies [29–32]. Through these studies, we have gained a wealth of information on the diverse molecular resistance mechanisms that tumor cells can adapt against precision targeted agents in cancer therapy. However, it is well-recognized that even complete responders to initial precision therapy with minimal to no detectable disease burden post-treatment ultimately will succumb to drug-resistant progression. This observation strongly argues for the presence of molecular minimal residual disease (MRD) upon initial remarkable tumor response. Hence, there is an unmet need to study drug resistance emergence during the early response phase of drug therapy within the spectrum of tumor evolution under therapeutic pressure. In this regard, the molecular mechanisms of drug resistance emergence and adaptive evolution of molecular MRD in responders remain poorly understood and ought to be aggressively investigated. Ultimately, these new insights of drug resistance and evolutionary changes during the course of therapy would allow us to devise rational therapeutic strategies and regimens to target the drug resistance driver events in the minimal residual cells as well as throughout the drug resistance evolution [33–35]. Due to the heterogeneous development of the tumor, minimal residual tumor cells can adopt a mutationally dependent or independent resistance against the drug to which most of the tumor responds. The goal of this review is to provide a critical appraisal of our current knowledge on tumor heterogeneity and its role underlying tumor incomplete response to precision therapy, leading to the emergence of minimal residual cells and early adaptive drug resistance. We attempt to summarize the gap in knowledge in understanding acquired resistance to early-phase lung cancer targeted therapy in partial and complete responders, and propose newly available technologies and methods to uncover the link between intratumoral heterogeneity and early adaptive drug resistance.

## Mechanisms of acquired drug resistance to lung cancer targeted therapy

**Fig. 1:**
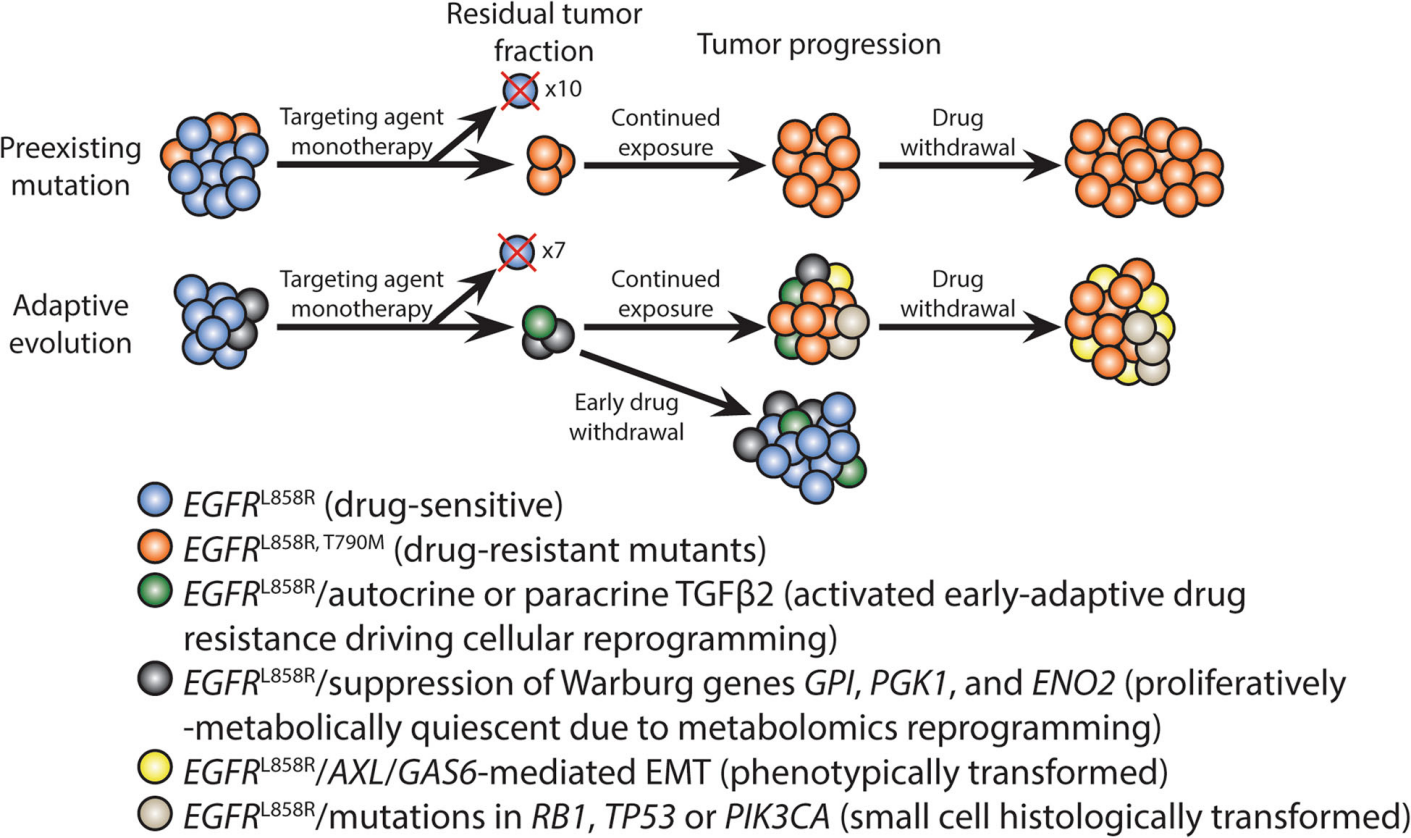
Models of drug resistance mechanisms following cancer targeted therapy. The *EGFR*-mutant model of drug resistance in lung cancer is shown here as an example. There are two recognized models of mechanisms of drug resistance known as preexisting mutations and adaptive evolution. In the preexisting mutations model, certain tumor cells growing within the parental population already have a survival advantage due to a preexisting mutation that can resist the targeting agent. Under continuous drug treatment, cells harboring the resistant mutation survive and proliferate to become the dominant clone, resulting in clinical drug resistance and tumor progression. Drug withdrawal at this point does not readily change the molecular makeup the cells. By contrast, in the adaptive evolution model, most tumor cells begin with a level playing field, with the exception of a subpopulation that may have been primed to activate prosurvival signaling pathways by an unknown regulatory or selective mechanism. While the majority of cells die under continuous drug treatment, a small subpopulation within the originally drug-sensitive cells will escape their initial dependence on the driver mutation, despite ostensibly identical genotype/genomic milieu, by adaptively altering either their transcriptome, signaling, or epigenome in a directed effort to survive against therapeutic pressure. This reprogramming process engenders the drug-escaping cells to enter into proliferative and metabolic quiescence. These adaptively resistant cells eventually acquire and accumulate mutations advantageous for further proliferative growth and the tumor progresses in fulminant resistance. In both aforementioned cases, the residual disease cells grow into a completely different tumor than the original under therapeutic pressure. However, previous work in vitro has demonstrated that early drug withdrawal can revert the adaptively resistant cells back to their parental, drug-sensitive state. This observation highlights the need for studying early adaptive resistant tumor cell populations and the mechanisms governing their shift to acquired resistance

**Fig. 2:**
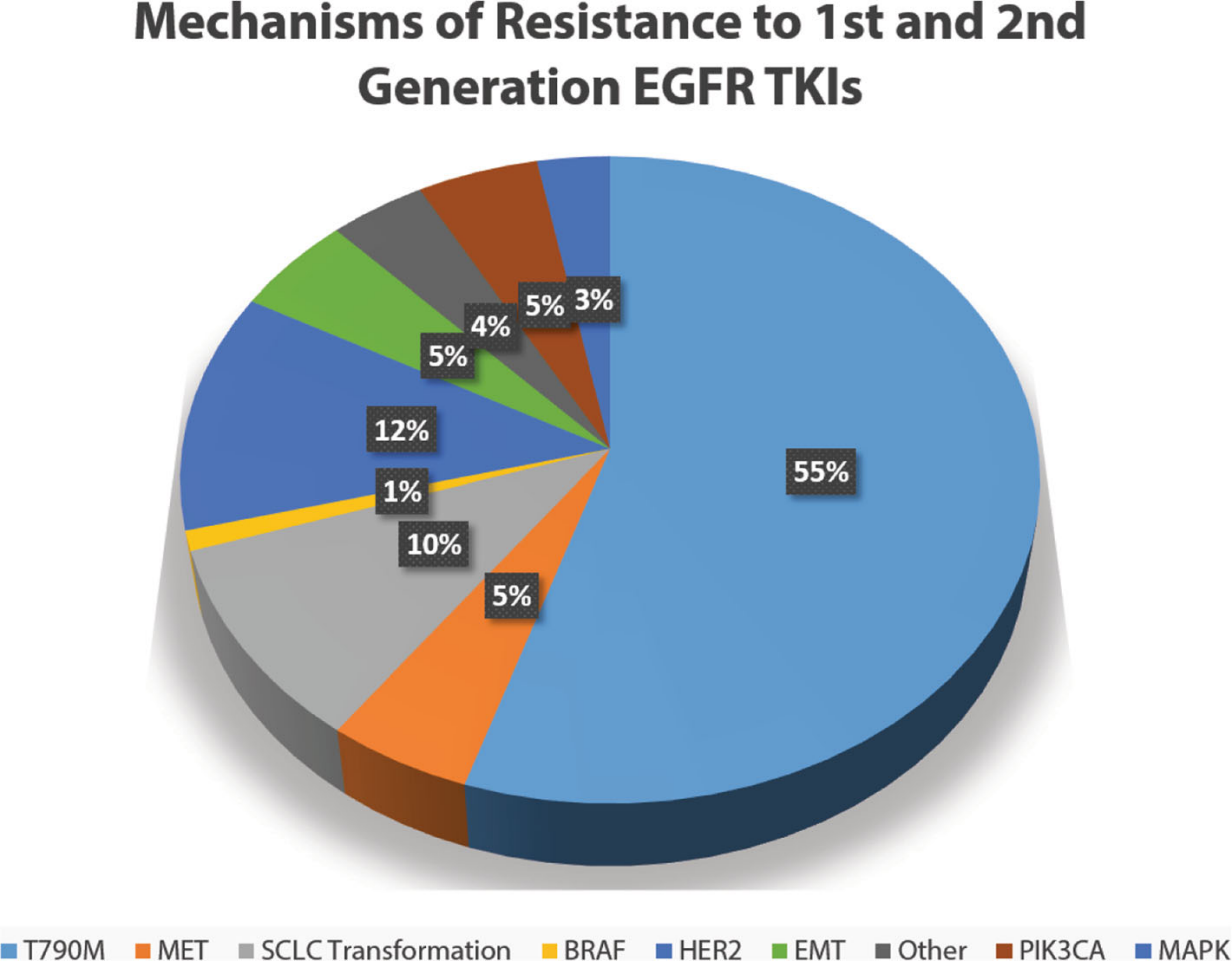
Landscapes of known molecular mechanisms of acquired targeted drug resistance to first- and second-generation EGFR-TKIs in lung cancer. The frequencies of each known mechanism are estimates acquired from studies based on tumor rebiopsies and repeat molecular tumor genotyping/genomic profiling at the time of acquired drug-resistant progression. The discovery of various mechanisms of acquired drug resistance further highlights the issues of tumor heterogeneity and adaptability of tumor cells to therapeutic pressure

Resistance to precision targeted therapy can be either preexisting or adaptive, which manifests clinically as primary and acquired drug resistance respectively. To this date, there are several well-accepted mechanisms of how acquired drug-resistant clones can emerge after initial treatment with precision targeted therapy (Fig. 1). We attempt to review here using the *EGFR*-mutant NSCLC targeted therapy as the key prototype model. First, tumor cells can have preexisting genetic alterations that confer drug resistance to the specific targeted inhibitor. For instance, the gatekeeper mutation substituting threonine for methionine at amino acid position 790 (T790M) in exon 20 of *EGFR* confers resistance to first- and second-generation EGFR-TKIs in 50–60% of *EGFR*-mutant NSCLC under TKI treatment (Fig. 2) [23, 29, 36]. Such resistance mutations may be preexisting but may also be adaptively acquired by a small subpopulation of cells during the course of tumor therapy and response. Previous work from two different teams provided evidence that the *EGFR* T790M mutation either enhances the affinity of the mutant kinase for ATP [37] or confers steric hindrance from the larger size of the methionine residue [38], although it is possible for both effects to occur in the same patient. Third-generation TKIs such as osimertinib, rociletinib, and WZ4002 have shown efficacy in counteracting the growth of *EGFR* T790M mutant tumors. The AURA2 phase II clinical trial for osimertinib demonstrated a 70% objective response rate for *EGFR* T790M-positive tumors [39], suggesting that we have a demonstrably effective method of controlling resistance as they emerge. Osimertinib was first approved by the U.S. Food and Drug Administration (FDA) as standard therapy for the treatment of *EGFR* T790M mutation-positive lung cancer [39–41]. Moreover, osimertinib has recently been further approved as first-line therapy for *EGFR*-mutant NSCLC expressing L858R or exon 19 deletion variant, based on the superior outcome when compared with the first-generation EGFR-TKIs (gefitinib or erlotinib) in the randomized phase III FLAURA study [42]. Osimertinib is now recommended by the National Cancer Center Networks (NCCN) as the preferred first-line option for treatment of *EGFR*-mutant NSCLC. Collectively, these preclinical and clinical research data suggest that resistance-conferring genetic alterations and their clinical emergence can be reasonably managed by subsequent improvement of the current targeted treatment to prevent or overcome drug resistance mutation(s), fueling strategies involving sequential monotherapies.

Nonetheless, the limitation of such an approach is apparent in that it represents a reactive approach of managing acquired clinical drug resistance. Drug resistance mutations against third-generation EGFR inhibitors have already been identified [43, 44], which often involves a substitution of the cysteine residue at position 797 to serine (C797S), thus blocking the covalent binding of these compounds to the mutated RTK. Interestingly, there is some heterogeneity in the mutation causing the amino acid substitution evinced by the two distinct possible mutations within the codon for amino acid 797, T → A and G → C, although the G → C mutation is more readily found in the plasma [43]. More intriguingly, recent studies have also shown that the allelic context of the C797S mutation further contributes to the heterogeneity in response to third-generation EGFR inhibitors [45]. In instances where C797S occurs on a different allele (in *trans*) than T790M, cells survive under third-generation TKI treatment but are susceptible to a combination of first- and third-generation TKIs [45]. By contrast, when the C797S mutation occurs on the same allele (in *cis*) as the T790M mutation, cells would be resistant to all EGFR-TKIs [45]. True to the concepts of sequential monotherapy, there are already efforts underway to develop inhibitors that can overcome the *EGFR* C797S mutant by targeting an allosteric site in a non-ATP competitive manner [46, 47]. Despite its reactive nature, applying sequential monotherapy strategies in *ALK*-rearranged NSCLCs resensitized tumors that were resistant to third-generation inhibitors, to first-generation inhibitors [48]. Although groundbreaking, it would be overly optimistic to label such strategy as the panacea for the quest of cancer cure since they fail to account for subsequent emergence of other forms of resistance. Sequential monotherapy strategies are limiting in that there is no foreseeable end to the recurring cycles of resistance emergence and development of newer generation drugs. This reactive treatment strategy remains limited in offering long-term survival impact in patients with advanced disease. There is an urgent need for a more proactive approach to identifying early molecular drivers of resistance in founder tumor cells in order to develop means to anticipate and combat their emergence preemptively.

Second, the network of signaling pathways within the tumor cell can be rather redundant with the inherent ability to confer “bypasses” in oncogenic signaling, resulting in incomplete suppression of the pathway targeted. Using the *EGFR* lung cancer model as an example, hyperactivation of the MET pathway either by amplification [49] or by increased receptor protein expression and phosphorylation [50] accounts for 5–10% of all patients resistant to EGFR-TKIs (Fig. 2) [29, 36]. It has also been shown to be a predictor of poor response to EGFR-TKIs regardless of the presence of sensitizing mutations [50]. Tumors with low initial levels of *MET* activation are predicted to show initial disease control (partial response or stable disease). However, initial disease control is then followed by a relatively short progression-free survival (PFS) because *MET* activation, although not as the initial preferred dominant pathway for cell proliferation and survival, can bypass the EGFR pathway for downstream signaling [36]. The percentage of cells containing MET pathway activation prior to EGFR-TKI treatment may determine whether the tumor cells present as intrinsic resistance or acquired resistance. *MET* amplification and overexpression of its natural ligand hepatocyte growth factor (HGF) [51] restores PI3K/AKT signaling, leading to resistance to EGFR-TKIs and expansion of preexisting *MET*-amplified cells [52]. HGF overexpression has also been correlated with T790M secondary mutation to confer resistance to irreversible EGFR-TKIs [53]. Because autocrine HGF-MET signaling has been previously shown to play a critical role in lung cancer progression [54] and co-overexpression of HGF with MET is not uncommon [55], it is attractive to propose targeting HGF-MET also as a potential strategy to curb resistance to EGFR-TKIs.

It should be noted, nonetheless, that *MET*-dependent resistance to EGFR-targeted therapy typically occurs in the late phase of adaptive drug resistance. *MET*-independent alternative mitochondrial-priming driven prosurvival signaling pathways bypassing targeted EGFR inhibition has been demonstrated, especially in the setting of adaptive drug-resistant escape during the early phase of treatment within days of drug initiation [34]. We demonstrated both in vitro and in vivo that the early drug-escaping persister cells had reactivated BCL-2/BCL-xL mitochondrial prosurvival signals and are more quiescence-like, displaying remarkably retarded cell proliferation and cytoskeletal functions. Inhibition of the mitochondrial BCL-2/BCL-xL prosurvival signaling in early drug persister survivor cells using BCL-2 homology domain 3 (BH3) mimetics such as ABT-737 or dual knockdown of BCL-2/BCL-xL was efficacious in eradicating these early adaptive drug persister cells. Interestingly, targeting BCL-2 alone using either RNAi-mediated knockdown [34] or the highly specific BCL-2 targeting BH3 mimetic ABT-199 [33] was not sufficient in eradicating the drug persister cells, supporting the notion of the importance of BCL-xL as the key survival factor in the drug persister cells. Studies also found adaptive reactivation of signal transducer and activator of transcription 3 (STAT3) within the minimal residual surviving drug persister tumor cells, which was co-activated with the SRC-YES-associated protein 1 (YAP1) pathway in *EGFR*-mutant NSCLC [56]. Inhibition of EGFR signaling simultaneous with paracrine or autocrine stimulation with TGFβ liberates cells from their dependency on EGFR for STAT3 activation, opting to activate the TGFβ-IL6-gp130-JAK2 axis instead. EGFR inhibition also results in ubiquitination of TRAF2 and subsequent nuclear translocation of NF-κB-RelA, which induces IL-6-mediated activation of the homodimerized nuclear STAT3. Co-targeting EGFR, STAT3, and SRC was also demonstrated to be synergistic in vitro as well as in vivo [56]. We propose to target the survival signaling machinery as the secondary “Achilles’ heel” in the early adaptive drug persister tumor cells in combination with EGFR-TKIs to more effectively eradicate the minimal residual drug persister tumor cells. An understanding of the crosstalk among different complementary pathways and the ability to reliably predict the resistance driver after inhibition of the primary resistance pathway is essential to control the emergence of drug resistance regardless whether it is at the early or late phase during treatment.

Third, histologic or phenotypic transformation of the lung adenocarcinoma subtype to small cell carcinoma has been observed in 3–15% of patients with clinically demonstrated acquired resistance to EGFR-TKIs (Fig. 2), including third-generation TKIs [23, 29, 36, 57–59]. Prior work in *EGFR* gene sequencing from repeat biopsies revealed that the *EGFR* activating mutation from the original adenocarcinoma remains in the SCLC cells that emerged during resistance [59], suggesting that these tumors have most likely undergone genuine phenotypic transformation from NSCLC to SCLC as opposed to developing drug-resistant SCLC de novo. The molecular mechanism of drug resistance via phenotypic transformation remains to be elucidated. It has been found that deletion of the retinoblastoma 1 gene (*RB1*) is common in SCLC [60]. Niederst et al. reported that *RB* loss was detected in 100% of the 10 SCLC-transformed *EGFR* mutants late in tumor progression, which is associated with increased neuroendocrine marker and decreased *EGFR* expression when compared with resistant NSCLC [59]. Interestingly, in line with our model of *MET*-independent EGFR-TKI drug escape, the resistant SCLC-transformed cancers demonstrated a similar increase in sensitivity to BCL-2 family inhibition. This transition is often accompanied clinically by a rapid acceleration in the growth rate, initial responsiveness to chemotherapy (especially SCLC regimen such as platinum-etoposide), and subsequent rapid clinical deterioration [36]. However, loss of *RB1* alone in vitro is insufficient to cause resistance or induce neuroendocrine differentiation. Concurrent somatic mutations in *TP53* and *RB1* are a classical characteristic of SCLCs and have been associated with primary resistance to EGFR-TKIs [61]. Considering the role of EGFR activity in promoting alveolar differentiation [62], it is possible that the progenitor pluripotent cells in vivo preferentially differentiate into NSCLC cells when EGFR is active. Under EGFR-TKI pressure, however, those same pluripotent cells may have accumulated additional genetic alterations (such as loss of *RB1* and *TP53*) and maintained a different epigenetic state to differentiate into an *EGFR*-independent lineage (such as SCLC). Hierarchical clustering analysis of RNA expression data revealed that cell lines derived from SCLC-transformed resistant biopsies are more similar to classical SCLC cell lines than to cell lines derived from resistant *EGFR*-mutant NSCLCs [59], suggesting that significant epigenetic and transcriptional changes have occurred during the transition. Moreover, drug sensitivity, genetic, and histologic profiling of the SCLC-transformed *EGFR* mutants further suggests that chronic EGFR inhibition can lead to the development of cancers that adopt a classical SCLC genotype and phenotype than other TKI-resistant cell states [59]. The lack of sensitivity to EGFR-TKIs could be explained by the low/absent EGFR expression compared with pre-resistant controls, a phenomenon that closely mimics SCLCs known to be able to grow and survive independent of EGFR expression or activation [63]. Together, research suggests that concurrent *TP53* and *RB1* loss can potentially transform lung cancer cells away from their NSCLC (adenocarcinoma) differentiation lineage roots and become more SCLC-like in an effort to resist continuous targeted drug treatment.

Another phenotypic transformation that can contribute to TKI resistance is the epithelial-to-mesenchymal transition (EMT) transdifferentiation program normally employed during embryonic development for tissue morphogenesis and development [64]. EMT was reported to be associated clinically with approximately 5% of EGFR-TKI acquired resistance cases (Fig. 2) [36], and was also observed with in vitro models of ALK-TKI drug resistance [65]. Induction of the EMT program is related to the activation of the AXL-GAS6 pathway [32, 66], the high co-expression of which has been shown to be an independent prognostic biomarker for poor survival in NSCLC patients with brain metastases [67]. AXL hyperactivation and evidence for EMT were previously reported in multiple in vitro and in vivo *EGFR*-mutant lung cancer models with acquired resistance to erlotinib independent of the *EGFR* T790M alteration and *MET* activation [32]. Moreover, genetic or pharmacological inhibition of AXL was shown to have the potential of drug resensitization to erlotinib in these tumor models. Individuals with *EGFR*-mutant lung cancers in acquired resistance to TKIs demonstrated increased expression of AXL and, in some cases, also of its ligand GAS6 [66]. Asiedu et al. demonstrated that pharmacological downregulation of AXL using MP470 (amuvatinib) has the potential to reverse EMT, attenuate self-renewal, and restore chemosensitivity of breast cancer cells that previously underwent EMT [66]. Expression of AXL was also correlated with expression of stem cell genes, regulation of metastasis genes, increase in tumorigenicity, invasion, and migration. Stable knockdown of AXL also led to downregulation of the NF-κB pathway and reduced tumor formation in vivo. Altogether, recent work has highlighted the association between EMT and drug resistance, and nominated AXL as an attractive targetable regulator of EMT to combat resistance.

More recently, adding to the knowledge of the mechanisms of acquired drug resistance, there is potentially a fourth mechanism described as “metabolic reprogramming” [33]. By analyzing the early adaptive drug-escaping cells using integrated transcriptomic and metabolomics profiling, it was discovered that cells in this state had increased plasticity mediated centrally by autocrine TGFβ2, similar to the pathway activating STAT3 as discussed earlier. The data suggested that plasticity is maintained through profound cellular adaptive “omics” reprogramming, including downregulation of key glucose metabolism regulatory Warburg genes (such as GPI, PGK1, and ENO2) and upregulation of the mitochondrial prosurvival marker BCL-2/BCL-xL. The early adaptive drug escape correlated with the cells being in proliferative-metabolic quiescence, susceptible to glutamine deprivation and TGFβ2 inhibition, and has enhanced EMT-ness and stem cell signaling. This study and others [68–70] further support a preemptive therapeutic co-targeting of bioenergetics and mitochondrial priming to suppress early drug escape emergence resulting from EGFR precision inhibitor, with this study specifically combining glutamine deprivation with broad BH3-mimetic to suppress early drug-escape emergence.

Overall, the predominant mechanisms of acquired drug resistance can be generally classified into the four groups aforementioned (Fig. 1). In particular, much has been uncovered in the domain of mutational and copy number alteration-related resistance, including but beyond *EGFR* T790M, *PIK3CA*, *HER2*, and *MET* (Fig. 2). Other remaining unknown mechanisms of acquired drug resistance have yet to be elucidated. With the advent of new genomics, transcriptomics, and proteomics technology, we can profile the mutational, epigenetic, and neoantigenic landscape of NSCLC in more details now than was ever possible in the past. The more proactive approach in achieving a deeper mechanistic understanding and unearthing new mechanisms of acquired drug resistance is to elucidate the emergence and evolution of MRD cells resulting from incomplete molecular response to therapy, which can continue to adapt and progress under ongoing therapeutic pressure and ultimately contribute to clinical tumor resistant progression.

## Understanding intratumoral heterogeneity in tumor evolution: the driving force behind minimal residual disease and drug tolerance-resistance

The goal of understanding and developing strategies to target minimal residual disease (MRD) is to potentially eradicate disease persistence and progression. MRD cells have been referred to as drug-tolerant “persister” cells due to their ability to persist in the lethal drug environment, or the “early adaptive drug-resistant” cells [33, 34] capable of escaping drug inhibition by activating prosurvival signaling pathways and adopting a reversible cell state similar to quiescence in order to maintain viability against drug adversities [71]. These “persister cells” or “early adaptive resistant cells” are able to emerge de novo even from single cell-derived, drug-sensitive populations [71], suggesting the early and dynamic nature of such a mechanism of resistance. Although the exact trigger for the conversion process from a drug-sensitive cell to a therapy-resistant cell is not completely understood, our studies and more recently by others suggest that the rapid, dynamic, and reversible emergence of drug persistence is an active form of early-phase “acquired” resistance, involving activated mitochondrial-prosurvival signaling activation, transcriptomic, and metabolomic reprogramming [33, 34]. The nomenclature “minimal residual disease” cells would be preferable as it accurately describes the nature and phenotype of these cells left behind in the therapeutic “battlefield” in a complete (or near-complete) responder, as “drug-escaping” or “drug-resisting” survivor cells. Of note, these cells are not merely passively tolerating the drug environment, but rather actively resisting or escaping the drug. Although emerging studies have highlighted the targetable molecular characteristics and cellular reprogramming involved in these drug-resistant survivor cells underlying the MRD, much is still not known about the molecular regulatory network that enables the emergence and evolutionary progression of these adaptive drug-resistant survivor cells.

**Fig. 3:**
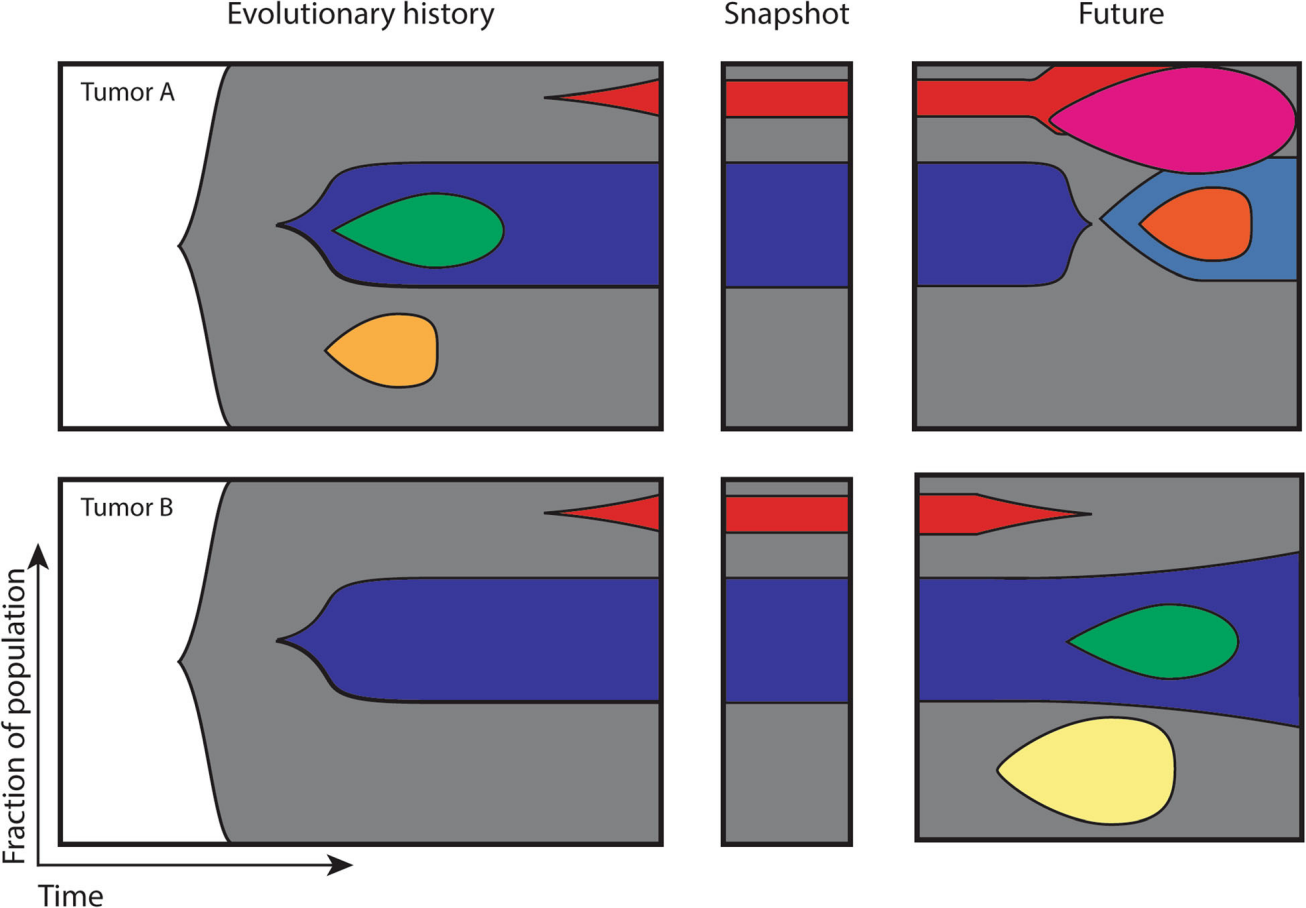
Spatial and temporal heterogeneity in tumor evolution. A single tumor tissue biopsy is equivalent to taking a mere “snapshot” of the molecular makeup of the tumor at a fixed time. The tumor’s evolutionary history and future as a result of progression and/or treatment would be missing from this single snapshot. Instead, serial and longitudinal tissue biopsies that track and follow the tumor’s development under therapy and during progression would empower more comprehensive and accurate representation of the tumor’s evolution, especially in exposing the conditions surrounding the emergence of subclones (as indicated by the different colors). Identification of subclones with known drug-resistant drivers can better inform the course of treatment most suitable for the tumor at its current state

The emergence of MRD can be attributed to branched tumor evolution and development, resulting in a number of subpopulations with different treatment response phenotypes than the original tumor-initiating cell. One way of modeling tumor evolution is by tracing multiple subpopulations of cells to their most recent common ancestor using a phylogenetic tree. The trunk of the tree represents clonal driver events that occur early in tumor development, whereas the branches represent subclonal driver events that differ from one subpopulation from another. It has been shown that a single ancestral clone can give rise to multiple subclones with [72] or without [73] treatment pressure throughout the course of tumor evolution. The branching evolution of clones is inherent with the phenomenon known as tumor heterogeneity. Broadly, tumor heterogeneity can be divided into two types: (1) intratumoral heterogeneity, which describes the co-existence of multiple subclones with distinct molecular profiles within a single tumor [74], and (2) intertumoral heterogeneity, which describes the molecular differences between tumors either from different sites in the same patient or from different patients entirely. Intratumoral heterogeneity can be further subclassified into spatial and temporal heterogeneity (Fig. 3). Due to various selection pressures, different tumor regions can have different drivers that appear to be clonal to the specific region [73, 75, 76]. As such, a single biopsy is only a small and limited sampling of the entire tumor, potentially leading to inappropriate generalization about the molecular constitution and driver of the entire tumor per se. Treatment with driver-specific targeted therapy then leads to incomplete therapeutic response. In the same way, a single snapshot of the molecular makeup of a tumor at a specific evolutionary time-point cannot reliably determine the full extent of tumor evolution and intratumoral heterogeneity. An understanding of the evolutionary history and future of tumors has the potential of revealing the most clinically significant subclones and common rules governing tumor evolution within and across cancer subtypes.

Intratumoral heterogeneity and tumor evolution are fueled by multiple factors, including genome doubling, mutational burden, and somatic copy number alterations. Multiregion sequencing studies in Caucasian NSCLC patients indicated high smoking-associated mutational burden for clonal mutations, resulting in low intratumoral heterogeneity [73]. Diversification occurs later on during tumor development and is primarily attributed to increased APOBEC activity that can be therapeutically targeted. Using similar methods, Nahar et al. found that intratumoral genomic heterogeneity in Asian lung adenocarcinoma patients, which are known to have low mutational burden, is characterized by high proportion of late subclonal mutations, early genome doubling events, and low copy number gains and losses [77]. The authors also noted the subclonal nature of high-amplitude copy number amplifications and deletions in their cohort. Additionally, tumors in the non-smoker cohorts were found to have a trend of acquiring less clonal co-driver mutations. Additional findings also suggest that *EGFR* mutations *per se* tend to be self-sufficient to initiate clonal expansion. As a result, lower clonal mutational burden was observed and whole genome duplication tends to occur earlier. Lower clonal driver mutational burden also correlated with better overall survival in patients [77]. These studies highlight the importance of elucidating the clinical trajectories undertaken by tumors by identifying the major factors contributing to intratumoral heterogeneity, and consequently its role in eventual emergence of MRD and ultimate treatment failure.

**Fig. 4:**
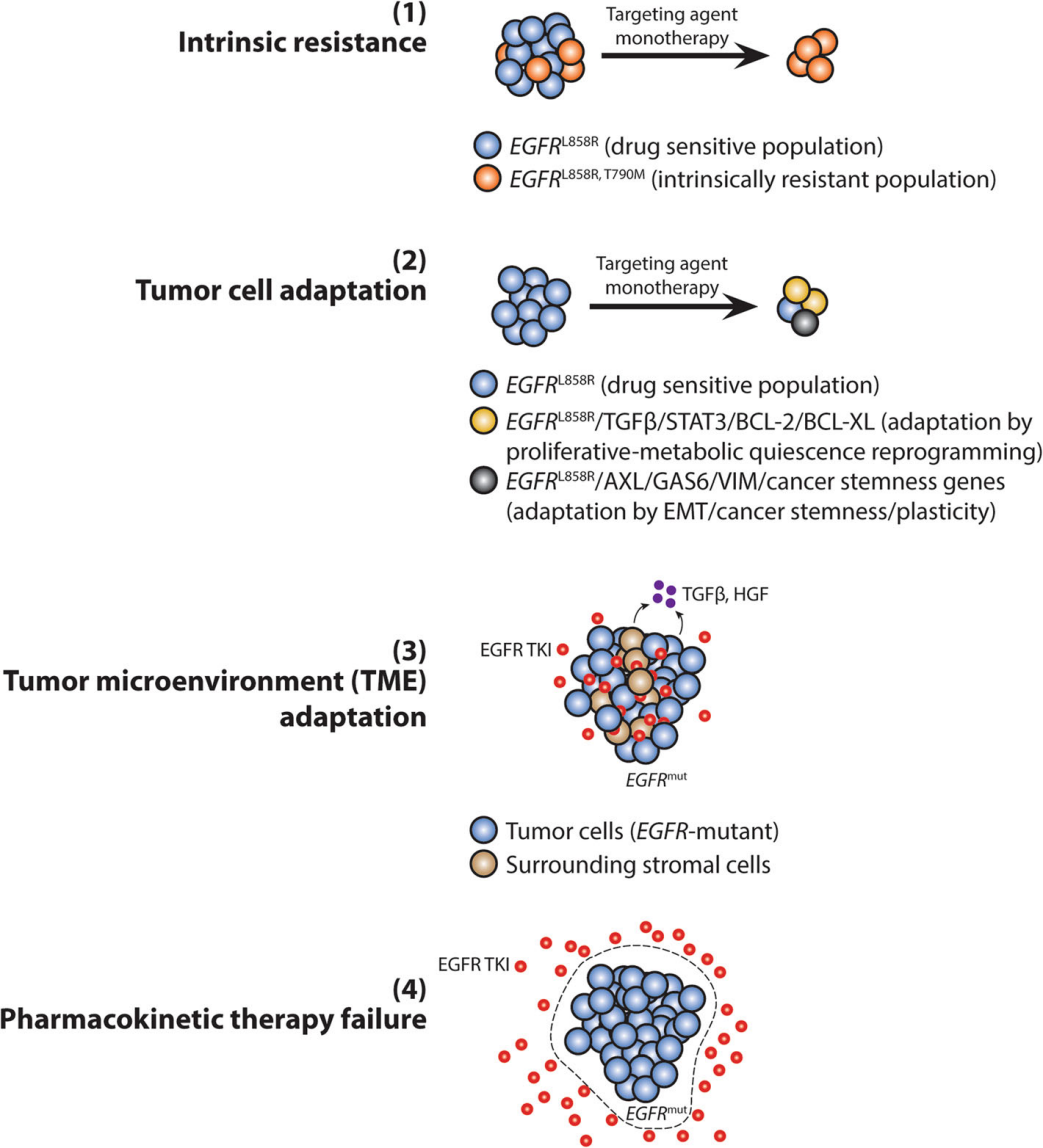
Conditions under which minimal residual tumor cells in molecular drug resistance can emerge. (1) Intrinsic resistance describes the cells’ inherent ability to resist the drug during initial therapy with preexisting stable genetic/genomic drug-resistant alteration(s). Shown are pretreatment lung adenocarcinoma cells harboring only the activating *EGFR* L858R mutation and cells that are double mutant for *EGFR* L858R and T790M. The T790M-mutants can survive initial treatments with an EGFR inhibitor (EGFRi) erlotinib or gefitinib, leading to incomplete response and eventual therapy failure and tumor progression stemmed from the expansion of the T790M clones. (2) Tumor cells adapt under therapeutic pressure to activate the early adaptive drug resistance program, engaging a cellular omics reprogramming scheme such as shift or modulation of prosurvival signaling, EMT-ness, cancer stemness and plasticity, glycolytic Warburg genes, among other undiscovered mechanisms. Drug-resistant molecular residual disease cells emerge as a result. As shown here in illustration, the STAT3/BCL-2/BCL-xL mitochondrial prosurvival signaling concurrent with hyperactivation of the TGFβ signaling pathway promote a drug-tolerant state that enables drug persistence during initial EGFR-TKI treatment. (3) The tumor microenvironment potentially contributes to the adaptive evolution of the tumor cells, resulting in minimal residual disease. As illustrated, stromal cells surrounding lung adenocarcinoma cells that secrete high levels of TGFβ have been known to stimulate the TGFβ axis in tumor cells via autocrine or paracrine signaling, granting them independence from EGFR signaling. TGFβ signals through IL-6, gp130, and JAK2 to stimulate STAT3 homodimerization. (4) Pharmacologic limitations, dose-limiting toxicities, or tumor intrinsic barriers can result in poor drug penetration into the tumor, resulting in pharmacokinetic therapy failure

Intratumoral heterogeneity can present as genetic or genomic [73, 78], epigenetic/epigenomic [79], neoantigenic/proteomic [80], metabolic/metabolomic [81], and tumor-microenvironment (TME) [82, 83] heterogeneity. Consequently, therapy-resistant residual disease cells may emerge through (1) intrinsic resistance, (2) tumor cell adaptive reprogramming, (3) tumor microenvironment (TME) adaptation, and (4) pharmacokinetic therapy failure [35] (Fig. 4). The factors of inter- and intratumoral heterogeneity affecting drug resistance are summarized in Table 1.

**Table 1:** Factors of inter-/intratumoral heterogeneity affecting drug resistance

Key biologic mechanisms	Molecular-phenotypic link to resistance	Drug resistance—intrinsic and acquired
**Genetic**	Tumor mutational burden	• Mutational heterogeneity and co-occurrence of different driver mutations that confer intrinsic resistance • Activation of bypass and redundant signaling pathway
**Genomic/Epigenomic**	Tumor adaptive molecular evolution/reprogramming	• Treatment-induced temporal and spatial driver mutational/non-mutational evolution • Acquired activation of bypass signaling pathways • Adaptively altered transcriptome • Therapy-induced secretome • Adaptively altered metabolome • Adaptive mitochondrial reprogramming
**Proteomic/Neoantigenic**		
**Metabolic/Metabolomic**		
**Tumor microenvironment**	TME and host interactions	• Increased availability of resistance-promoting ligand whether intrinsic of the stromal cells or influenced by tumor cell secretions • Heterogeneous development of physical or stromal barriers to drug penetrance • Heterogeneous organ-specific stromal milieu providing different drug-protective mechanisms to tumor cells • Pharmacokinetic failure from differential exposure to therapy

Intrinsic resistance can arise as a result of heterogeneous stable genetic alterations either preexisting on the target oncoprotein resulting in a drug-resistant mutant form, or on a different signaling molecule activating a complementary pathway to bypass signaling. Turke et al. identified subpopulations of cells with *MET* amplification within *EGFR*-mutant lung cancers prior to drug treatment [52], which contributes to gefitinib resistance when activated by HGF through a PI3K/AKT/GAB1 signaling pathway. The authors demonstrated the ability to select *MET*-amplified *EGFR*-mutant cells when the parental cell population was briefly treated with HGF. Intriguingly, low level *MET* amplification was observed even in populations derived from single cell clones from the parental drug-naïve cell population, suggesting that some tumors are predisposed to maintain heterogeneity even in the absence of therapeutic pressure [52]. Additionally, acquired resistance can develop through the tumor’s heterogeneous response to therapy, with some subpopulations adopting a quiescence-like cell state, altering their signaling, secretome, transcriptome, and metabolome in the process [33, 71, 84–86]. In support of a therapy-induced altered cell state described earlier involving transcriptome and metabolome changes [33], Obenauf et al. demonstrated an altered and complex network of secreted signals in BRAF, ALK, or EGFR TKI-treated melanoma and lung adenocarcinoma cells [86]. The therapy-induced secretome was shown transcriptomically to consist of more than 5000 up- and downregulated secreted factors, significantly overlapping with the gene expression changes of their in vivo model, that are released into the tumor microenvironment stimulating both tumor cells and the surrounding stromal cells. Increased tumor proliferation induced by the secretome was associated most prominently with activation of the AKT pathway, and dual inhibition of the RAF and the PI3K/AKT/mTOR signaling pathways reduced the growth of drug-resistant cells in a *BRAF*-mutant melanoma model [86]. Heterogeneity in the surrounding stroma that constitutes the TME may also be substantial in influencing treatment response of tumors at different sites (i.e., primary tumor vs. metastatic tumor). For instance, in melanoma patients, increased secretion of HGF from surrounding stromal cells increases MET pathway signaling in melanoma cells, resulting in resilience against BRAF targeted inhibitors [87, 88]. In addition, there can be disparate development of physical and stromal barriers that restrict effective drug delivery to cells as well as drug efflux pumps that vary in concentration and activity across cells, resulting in inadequate delivery of drugs to have any meaningful impact on the intended target [89, 90]. All the aforementioned factors of heterogeneity enable drug escape and resistance against precision therapy and survival during targeted inhibition. The nature, degree, and extent of upfront tumor heterogeneity may determine if there will be measurable residual disease after initial drug response in cases of more substantial driver genomic heterogeneity in a patient.

Conceivably, in a highly oncogenic addicted tumor, the emergence and establishment of non-measurable MRD after initial remarkable treatment response should be expected. This is because at the time of maximal response, intratumoral genomic heterogeneity among the residual tumor cells should be understandably less pronounced, consisting mainly of adaptive drug persister cells of similar genotypes and highly conforming transcriptomes. In an in-depth analysis of the transcriptional dynamics during patient-derived primary oral squamous cell carcinomas (OSCC) cell lines evolution, Sharma et al. set out to explore if there is a difference in the set of mechanisms by which tumors acquire resistance to cisplatin given that they are phenotypically homogeneous or phenotypically heterogeneous pretreatment [91]. It was observed that both phenotypically homogeneous and heterogeneous tumors each go through four distinct stages of evolution, diverging at the second stage. For phenotypically heterogeneous tumors, preexisting cells from the drug-naïve population (stage 1) completely overwhelm the culture by 6 weeks of cisplatin treatment (stage 2), exhibiting the classic example of overt intratumoral heterogeneity-mediated clonal selection that favors selection of preexisting cancer stem-like cells; for phenotypically homogenous tumors, de novo transdifferentiation into a drug-resistant cell population (stage 2) is achieved via epigenetic SOX9-associated mechanisms under drug-selection. The now predominant cell type expands (stage 3) into the metastasis-prone, drug-resistant population (stage 4). Altogether, preexisting ITH leads to the selection of cancer stem-like cells under selection pressure, whereas stress-induced transdifferentiation drives homogenous cell populations to evolve adaptively to convergent phenotypic states that are predetermined by a poised bivalent epigenome.

It is difficult to ascertain whether MRD, as a result of intratumoral heterogeneity within treated tumors, exhibits intrinsic or acquired resistance as these resistance terminologies themselves are closely associated with clinical response (intrinsic resistance defines lack of response to initial drug treatment, whereas acquired resistance defines resistance resulting in disease progression following initial response). It is also difficult to delineate whether these heterogeneous genomic mutations contributing to MRD are preexisting or adaptively evolved, as MRD cells with adaptively evolved mutations must have some preexisting features/characteristics that predispose them to embark on an evolutionary path into specific drug-resistant mutations/alterations. However, based on available data thus far, one may justifiably say at this time that adaptive emergence of persister cells is primarily non-genetic in nature at least at the outset.

For many years, clinical researchers in hematologic malignancies have been measuring levels of MRD after cancer therapy, as an indicator of treatment efficacy [92–94]. This quantification of MRD is known as “depth of response” (DepOR) and can be accurately measured using sensitive methods such as flow cytometry and next-generation sequencing. In solid tumors, DepOR is defined as the maximum percentage of tumor shrinkage from baseline observed in a patient. Due to traditional clinical endpoints such as PFS and overall survival (OS) having a longer time to maturity, surrogate endpoints such as overall response rate (ORR) and duration of response (DoR) are commonly used to determine treatment efficacy early on during treatment. Nonetheless, ORR may be limited in representing treatment efficacy as it is a static measurement of the percentage of patients with reduction in tumor burden of a predefined amount, and it dichotomizes patients into responders and non-responders based on the Response Evaluation Criteria In Solid Tumors (RECIST). This drawback of ORR is most evident in the FLAURA trial, in which although the ORR in patients with *EGFR* activating mutations was highly comparable between the osimertinib cohort and the standard-of-care first-generation EGFR-TKI cohort (80% vs. 76%), the median PFS was significantly longer with osimertinib than with first-generation EGFR-TKIs (18.9 months vs. 10.2 months) [42]. On the other hand, DepOR was a better predictor of this difference in PFS, since the authors noted that the median best percentage change in target-lesion size (maximum decrease from baseline, or minimum increase from baseline in the absence of a decrease) was less in the osimertinib group versus the standard EGFR-TKI group (− 54.7% vs. − 48.5%, *P* = 0.003). Compared to ORR, DepOR has a shorter time to maturity, is a serial and continuous measure of response, and maintains a more granular, patient-to-patient view of magnitude of response (instead of grouping patients into responder vs non-responder). Previous reports have demonstrated that DepOR is significantly associated with both PFS and OS in NSCLC [42, 95–97], metastatic colorectal cancer [98–103], and gastric cancer [104].

## Previous limitations and novel methods for linking intratumoral heterogeneity and drug resistance

Intratumoral heterogeneity not only enables the survival of residual disease cells that eventually is the cause of a more aggressive tumor relapse but also serves as the impetus of the failure of single agent targeted inhibitors to induce long-lasting durable response and survival benefits despite initial remarkable tumor response. Next-generation targeting agents, while able to inhibit mutant drug-resistant forms of the intended target, have also been shown to elicit incomplete therapeutic response. The most prominent example of the inadequacy of next-generation targeted inhibitors in curbing eventual disease progression is in the case of the third-generation EGFR inhibitor osimertinib in *EGFR* T790M mutated tumors [39]. As previously described, the T790M mutation in *EGFR* renders cells resistant to first-generation inhibitors. While response to osimertinib in genomics-matched patients is often remarkable, acquired resistance develops even earlier (overall median PFS = 8.2 months) [105] than in erlotinib-treated tumors with *EGFR* activating mutations (overall median PFS = 9.7 months) [106]. A similar decrease in the time-to-progression is also observed for *ALK*-rearranged NSCLC (crizotinib overall median PFS = 8.0–10.0 months [107–109], ceritinib overall median PFS = 7.0 months [110]). Nevertheless, these observations can be attributed to the fact the patients in these studies were previously treated with at least one line of therapy, and that the tumor generally have been more heterogeneous at the time of treatment to overcome acquired drug resistance already established. First-line osimertinib treatment in advanced NSCLC with mutant *EGFR* yielded an overall median PFS of an impressive 19.3 months in the phase I study [40]. Most recently, similar observations were confirmed in the phase III clinical trial, revealing that osimertinib first-line treatment led to a similar response rate compared to the first-generation EGFR-TKIs (80% vs. 76%), but resulted in a significantly superior PFS (18.9 months vs. 10.2 months) [42]. It is tempting to postulate that the preemptive use of osimertinib as first-line TKI in *EGFR*-mutant NSCLC patients resulted in not only prevention of *EGFR* T790M mutation emergence but also a “deeper molecular response” in the oncogene-addicted tumor cells. These clinical evidence arguably lends further support to the concept of a “preemptive” targeted inhibition being more superior to a “reactive” sequential targeted therapeutic approach. With what is currently known regarding MRD, this remarkable delay in drug resistance development could be further enhanced by targeting residual disease drivers simultaneously in the form of rational polytargeting combinational therapy.

Rational upfront polytargeting therapy has the potential to induce a more complete and durable tumor response than monotherapy due to the ability of the former to address not only the tumoral heterogeneity issues but also the multifaceted nature of MRD. To overcome resistance to genotype-matched targeted therapy, rational polytargeting therapy to target the primary addictive oncoprotein driver as well as signaling molecule(s) in the drug escape/resistance-conferring pathway can be tested as first-line treatment or sequentially to first-line treatment failure. Understandably, in many cases, second-line polytargeting therapy demonstrated low efficacy due to the prior establishment of drug resistance, which might already be heterogeneous in nature, post first-line therapy failure [111–114]. On the other hand, using rational polytargeting therapy as a first-line therapeutic strategy prior to molecular drug persistence/resistance emergence conceivably can serve as an effective restraining barrier against drug resistance associated with targeted monotherapy. This is also exemplified in the case of combining BRAF inhibitors with MEK1 inhibitors in overcoming bypass signaling of the RAF-MEK-ERK signaling cascade in *BRAF* V600E mutant melanoma [115, 116]. This strategy has now been further adopted and approved for use in *BRAF*-mutant lung cancer recently [117–119]. First-line BRAF-MEK inhibitor combination therapy improved patient survival compared with first-line BRAF inhibitor monotherapy, consistent with the hypothesis that targeting residual tumor cells can prevent eventual tumor progression [120–122]. These studies demonstrate the importance of timing in the administration of polytargeting therapy to effectively control residual disease and drug resistance. Despite promising better responses, rational polytargeting therapies could still be limited and challenging due to increased risk of adverse events compared with monotherapy [120–122]. Nonetheless, this could be at least partly alleviated by optimized drug design and development of drugs that have improved therapeutic window with more potent and specific target efficacy and less off-target adverse effects. A recent example of successful combination therapy is seen in the IMpower 150 study, in which atezolizumab, bevacizumab, carboplatin, and paclitaxel (ABCP) were administered in combination in treatment-naïve patients with metastatic nonsquamous NSCLC [123]. Both overall survival and progression-free survival were significantly improved compared to standard-of-care with similar safety risks. This study also proved effective as first-line therapy regardless of PD-L1 expression and *EGFR* or *ALK* genetic alteration status. In particular, it was found to be effective as a treatment strategy for the targeted therapy resistant patients with *EGFR* mutation or *ALK*-rearrangement. The chemo-immunotherapy combined with antiangiogenesis therapy is thought to impact the TME in enhancing the PD-L1 immunotherapy efficacy as the underlying mechanism of action. Furthermore, it has also been recently reported that the ABCP combination therapy could induce a remarkable complete response even after merely one cycle of treatment in heavily pretreated *EGFR*-mutant lung adenocarcinoma that progressed through erlotinib and osimertinib in targeted drug resistance [124]. Efforts to combine agents that not only target non-overlapping mechanisms of resistance but also elicit fewer adverse events are warranted, and should be guided by an understanding of the residual disease state in selection of agents and measurement of efficacy in polytargeting therapies [125].

As illustrated earlier, the understanding of MRD is inseparable from understanding intratumoral heterogeneity. Recently developed techniques allow for more in-depth studies of spatial and temporal heterogeneity within a single tumor. In addressing spatial heterogeneity, multiregion whole-genome and whole-exome sequencing methods [73, 126] have been employed to overcome the issue of limited sampling of a tumor in cancer genomics analysis. The TRACERx study conducted whole-exome sequencing of multiregional biopsies from a single tumor (at least 0.3 cm to 1.0 cm apart) in resected stage I to III NSCLC patients, and demonstrated the mutational and copy number differences between regions of a single tumor [73]. It was found that chromosomal instability contributed to the acquisition of heterogeneous subclonal driver mutations and copy number alterations later in tumor development. Driver mutations in *EGFR*, *MET*, *BRAF*, and *TP53* were found almost always clonal in lung adenocarcinomas, whereas alterations in *PIK3CA*, *NF1*, genes involved in chromatin modification, and DNA damage response and repair occurred later in tumor evolution. These studies suggest that the detection of specific mutations in single biopsies may not reflect the profile of the tumor as a whole. The study of tumor evolution throughout the course of treatment using the methods described above has the potential to elucidate biomarkers associated with treatment response and acquired resistance.

One disadvantage of multiregion sequencing is the need for multiple biopsy sampling, which is impractical and undesirable in real-life patient care scenario particularly in advanced stage diseases [127]. To this end, liquid biopsies coupled with molecular profiling have gained much momentum in recent years. Liquid biopsy can be quite beneficial since it is less invasive compared with traditional tissue biopsies and is able to provide a more comprehensive tumor profile presumably with better representation of tumor heterogeneity [128–130]. Generally, liquid biopsy involves isolating circulating tumor cells (CTCs) or circulating tumor DNA (ctDNA) from blood samples and subsequently conducting molecular, genomic, and proteomic assays to obtain a holistic profile of the tumor. Currently, clinically adapted liquid biopsy typically involves plasma-based ctDNA assays using next-generation sequencing in genomic mutation or copy number determination. In 2016, the U.S. FDA approved the Cobas *EGFR* Mutation v2 Test as an in vitro companion diagnostic for the detection of exon 19 deletions, exon 21 L858R substitution mutations, and T790M mutations from plasma samples [131, 132]. The approval was based on the ENSURE study, a multicenter, open-label, randomized phase III study to evaluate the efficacy and safety of erlotinib versus gemcitabine plus cisplatin as first-line treatment for stage IIIB/IV NSCLC patients [133]. Plasma tested positive for *EGFR* mutations in 76.7% of tissue-positive specimens, and tested negative in 98.2% of tissue-negative specimens. The approval of the Cobas test prompted multiple investigations including those that studied plasma ctDNA for early prediction of response to TKIs [134], for detection of *EGFR*-T790M in previously EGFR-TKI-treated NSCLC patients with disease progression [135], and for the development of AZD9291 (osimertinib) [105, 136]. One study investigated the eligibility of previously treated NSCLC patients for osimertinib by testing for the presence of the T790M mutation in their plasma [135]. Although plasma tests only moderately agree with tissue tests (61% positive, 79% negative), comparing plasma tests with next-generation sequencing yielded positive and negative agreement rates of 90% or higher. In addition, tumor burden [137] and tumor mutational load [138, 139] assays are being developed in this arena as an indication of treatment response and as a potential predictive biomarker for immunotherapy, respectively. As powerful as liquid biopsy promises to be, it is limited by intertumoral heterogeneity. More specifically, the inability to trace the source of the ctDNAs, resulting in the possibility of confounding downstream analyses due to intertumoral heterogeneity. Garcia-Saenz et al. found that although plasma *PIK3CA* mutation levels correlated with treatment response in most advanced breast cancer patients in their cohort, the treatment response discordant rate was as high as 25% (2/8 patients), with the discordance being attributed to differential drug sensitivity within the metastatic tumor [137]. As previously discussed, single time-point biopsy, whether as tissue or plasma samples, provides limited information about the tumor’s evolutionary history and future. To overcome this issue, serial longitudinal biopsies can be done to analyze changes in the tumor with or without therapeutic pressure. Due to the relative ease on patients, liquid biopsy is gaining much momentum as a more preferable method for longitudinal monitoring of tumor evolution.

Single-cell molecular analysis is becoming increasingly important in uncovering clonality and reconstructing the evolutionary lineage of a tumor. Bulk analyses aggregate results from multiple cells from a sample and run the risk of missing vital information from rare cell subpopulations [140]. Using single-cell techniques, Lawson et al. demonstrated that the subpopulation of metastatic breast cancer cells are unique in their increased expression of EMT, stem-like, prosurvival, and dormancy-associated genes [141]. Much like cells with metastatic potential, MRD cells are rare subpopulations within drug-sensitive tumors that often drive disease progression. Rambow et al. demonstrated the feasibility of using a combination of fluorescence-based and microfluidics-based capture techniques to study and target the driver of MRD in melanoma exposed to concurrent RAF/MEK inhibition [142]. The authors identified a transcriptional program associated with neural crest stem cells in the minimal residual melanoma cells driven by the nuclear receptor RXRG and showed that targeting RXR signaling synergizes with targeted therapy to delay time to disease progression. Recently developed single-cell proteomics methods allow for multiplexed protein detection from single cells and analyzing functional protein expression simultaneously with gene expression [143, 144]. However, single-cell analyses lack in their ability to recapitulate the effects of cell-cell and cell-matrix interactions, as the tumor has to be dissociated prior to conducting these experiments. Nonetheless, the higher resolution of single-cell methods and the ability for multiplexing promise early identification of resistance drivers and aid in the development of rational polytargeting therapies that can preemptively prevent tumor progression driven by residual disease cells.

## Molecular profiling for tumor-agnostic, driver-specific targeted therapy

Following the advent and clinical adoption of EGFR-targeted therapy in *EGFR*-mutant NSCLC, a growing list of additional genomically matched targeted therapies continue to emerge in the treatment of various solid cancers including lung cancer. These include therapeutics that target specifically addictive oncogenic alterations as in *ALK*-translocations [145–148], *ROS1*-translocations [148–150], *RET*-translocations [151–153], *BRAF* mutation [154–156], *MET* amplification and *MET* exon 14 skipping mutations [157–160], and most recently *NTRK*-translocations [161]. Emerging data suggest that the precise molecular mechanisms of drug resistance, and the spectrum of such mechanisms could be different among different molecular targets and their underlying targeted therapeutics. As previously described, the dominant resistance mechanism for *EGFR*-driven first- and second-generation TKI-treated NSCLCs is acquisition of the T790M mutation (Fig. 2). On the other hand, *ALK*-driven TKI-treated NSCLCs have a different pattern of drug resistance mechanisms, with all mutations in the *ALK* gene accounting for approximately 28% [162], and no dominant gatekeeper mutation is as frequently seen as in *EGFR* T790M. Other ALK-TKI resistance mechanisms can be further classified based on whether or not the tumor is still dependent on ALK signaling (ALK^+^/ALK^−^). These ALK^+^ and ALK^−^ resistance mechanisms are approximately equally prevalent [162]. Most interestingly, recent studies uncovered a new paradigm of various oncogenic fusions such as *CCDC6*-*RET* as part of the genomic landscape of osimertinib acquired resistance mechanisms [163]. Other osimertinib resistance genomic alterations recently reported include *EML4*-*ALK*, *MET* amplification, *KRAS* mutations, *BRAF* mutations and *PIK3CA* mutations, and *PTEN* deficiency [164].

Most recently, Drilon et al. reported on an integrative analysis of three phase 1–2 studies evaluating the efficacy of larotrectinib (also known as LOXO-101)—a highly selective small-molecule pan-TRK inhibitor—in 17 unique *NTRK* fusion-positive cancers in 55 adult and pediatric patients [161]. Overall response rate was reported to be between 75 and 80%, with 71% responses ongoing and 55% patients remaining progression-free after 1 year. Despite the durable responses, it is reasonable to expect the ultimate emergence of acquired resistance to TRK-targeting agents as has been previously reported separately in two patients treated with the multikinase inhibitor entrectinib, which has activities against *NTRK*, *ROS1*, and *ALK* [165, 166]. These tumors acquired resistance mutations affecting the kinase solvent front and xDFG motif, which interferes with larotrectinib and entrectinib binding directly. Further functional studies have confirmed that these mutations confer resistance to all TKIs with activity against TRK [167, 168]. With this knowledge, a second-generation TRK-TKI, LOXO-195, has been newly designed to overcome acquired resistance mediated by recurrent kinase domain (solvent front and xDFG motif) mutations [169]. LOXO-195 was shown to possess potent and selective activity against all three TRK kinases, their fusions, and acquired resistance mutations identified both in preclinical models and in patients. The development of LOXO-195 introduces the exciting potential of further strategies to prevent or overcome acquired resistance to first-generation TRK-TKIs, extending the duration of response and long-term survival in a tumor- and age-agnostic fashion. As such, the recent tumor-agnostic FDA approval of larotrectinib in *NTRK* fusion-positive diverse solid tumors regardless of tumor type origin unveils a new era and novel paradigm of molecular-genomics precision medicine. Furthermore, the emergence of larotrectinib in *NTRK* fusion-positive tumors including lung cancer now strongly validates the crucial importance of upfront unbiased broad and comprehensive tumor molecular-genomic profiling in order to optimize therapeutics decisions for lung cancer patients. While there may still be some room for debate as to what constitutes the best platforms for tumor molecular profiling, it is now widely accepted that next-generation sequencing based profiling platform would be regarded as ideal to enable tissue- and time- as well as possibly cost-efficiency in such an essential endeavor for modern personalized cancer medicine.

## Conclusion

In summary, acquired drug resistance to targeted therapy begins with the emergence of drug-tolerant MRD cells. Clonal studies of tumor evolution have proven to yield novel and important information regarding apparently similar tumors classified based on driver mutations alone. This is true because factors contributing to intratumoral heterogeneity such as mutational burden, genome doubling, and copy number alterations can determine the evolutionary path of the tumor, and consequently, the mechanism of drug tolerance and early drug resistance. Such studies have been made possible due to the recent availability of multiregion exome sequencing, among other advances in genomics techniques and NGS platforms, which takes into account the presence of different subclones in a tumor that are largely driven by the same driver mutation. Further research is needed to be conducted in more advanced stage cancers. In this regard, emerging advance liquid biopsies technologies, especially when performed in serial longitudinal setting during treatment, can be considerably attractive due to its non-invasive nature and the ability to overcome at least partially the confounding challenges of tumor heterogeneity in a patient. Liquid biopsies could potentially discern and detect subclonal cell populations within a tumor with reasonable sensitivity and specificity. Moreover, recently developed single-cell harvesting and genomics-bioinformatics analyses methods of cells undergoing targeted drug treatment allow for studying targetable drivers of MRD. Future evolutions of precision medicine could involve preemptive combinatorial targeting of MRD drivers as well as tumor drivers.

## Data Availability

Not applicable.

## Abbreviations

ALK: Anaplastic lymphoma kinase
BH3: BCL-2 homology domain 3
CTC: Circulating tumor cells
ctDNA: Circulating tumor DNA
DepOR: Depth of response
DoR: Duration of response
EGFR: Epidermal growth factor receptor
EMT: Epithelial-to-mesenchymal transition
FDA: Food and Drug Administration
HGF: Hepatocyte growth factor
MRD: Minimal residual disease
NCCN: National Cancer Center Networks
NSCLC: Non-small cell lung cancer
ORR: Overall response rate
OSCC: Oral squamous cell carcinoma
PFS: Progression-free survival
RB1: Retinoblastoma 1
RECIST: Response Evaluation Criteria In Solid Tumors
RTK: Receptor tyrosine kinase
STAT3: Signal transducer and activator of transcription 3
TKI: Tyrosine kinase inhibitor
TME: Tumor microenvironment
TRK: Tropomyosin receptor kinase
YAP1: YES-associated protein 1
